# Digital Psychiatry in the COVID-19 Era: the First Italian Cross-National Survey

**DOI:** 10.1192/j.eurpsy.2022.1467

**Published:** 2022-09-01

**Authors:** L. Orsolini, S. Tempia Valenta, V. Marchetti, S. Bellagamba, V. Salvi, U. Volpe

**Affiliations:** 1 Unit of Clinical Psychiatry, Polytechnic University of Marche, Department Of Neurosciences/dimsc, Ancona, Italy; 2 School of Medicine, Polytechnic University of Marche, Dimsc, Ancona, Italy

**Keywords:** digital psychiatry, telepsychiatry, psychiatry training, education

## Abstract

**Introduction:**

The COVID-19 pandemic led to the implementation of digital psychiatry (DP) in everyday clinical practice, resulting in the need for a skilled healthcare workforce.

**Objectives:**

Our purpose was to investigate the level of training, knowledge and expertise of young mental health professionals and medical students in DP, as well as exploring their beliefs and experiences in this field.

**Methods:**

An *ad hoc* cross-sectional survey was designed and administered to Italian medical students, psychiatry trainees, and early career psychiatrists.

**Results:**

Most of the sample declared that the topic of DP was never discussed within their academic training (89.1%), mainly revealing an overall lack of knowledge on DP. Nevertheless, they mostly declared that DP represents a valuable therapeutic tool in mental health (80%) and that the academic background should include a dedicated course/module (54.4%). Moreover, most subjects declared that DP is less effective than in-person interventions (73.2%), despite the emerging evidence that being trained in DP is significantly associated with the belief that digital and in-person interventions are comparable in their effectiveness (p≤0.05).

**Conclusions:**

During the current pandemic, DP represented an ideal response to the need for physical distancing by ensuring the advantage of greater access to care. However, DP interventions are still uncommon, and there remains a certain resistance to their use in mental health care. The lack of formal training during the academic training could be a limiting factor. Therefore, addressing these issues in academic settings could be crucial to spreading this innovative practice also in the post-COVID-19 era.

**Disclosure:**

No significant relationships.

